# Distribution and morphologic characterization of telocytes in rat ovary and uterus: insights from ultrastructural and immunohistochemical analysis

**DOI:** 10.1007/s00418-024-02313-w

**Published:** 2024-07-30

**Authors:** Merjem Purelku, Hakan Sahin, Gozde Erkanli Senturk, Gamze Tanriverdi

**Affiliations:** 1 Institute of Graduate Studies, Istanbul University-Cerrahpasa, Istanbul, Turkey; 2 Department of Histology and Embryology, Istanbul University-Cerrahpasa, Istanbul, Turkey

**Keywords:** Female reproductive organs, Stromal cells, Transmission electron microscopy

## Abstract

Telocytes (TCs) are characterized by a small oval-shaped cell body with long prolongations that are called telopods (Tps). PDGFR-β and c-kit markers may assist for the immunohistochemical identification of TCs; however, by these means they cannot be identified with absolute specificity. Transmission electron microscopy (TEM) is considered as a gold standard method for TCs observation. Studies on TCs in the female reproductive system are limited, and there is a lack of awareness regarding TCs in rat ovaries. We aimed to demonstrate the existence and morphology of TCs in rat ovaries, alongside previously studied TCs in rat uteri. Thus, ovaries and uteri from young adult Sprague–Dawley female rats (*n* = 8) with regular estrous cycles were collected. Then, left ovaries and uteri were proccessed for TEM analysis, while the right ones were used for immunohistochemistry. As a result, TCs were seen throughout the rat’s ovarian stroma with their characteristic cell bodies, Tps, podomes (Pds) and podomers (Pdms). Tps were situated within the thecal layer of the follicles, surrounding the corpus luteum and blood vessels. Ovarian TCs were recognized to have relationship with other TCs/stromal cells. Subsequently, TCs were seen in stroma of endometrium with surrounding blood vessels and uterine glands, myometrium and perimetrium in rat uteri. There was also no statistical significance between the number of c-kit+ and PDGFR-β+ telocyte-like cells in both rat ovarian (*p* = 0.137) and endometrial stroma (*p* = 0.450). Further investigation of the roles and functions of TCs in the female reproductive system is needed.

**Supplementary Information:**

The online version contains supplementary material available at 10.1007/s00418-024-02313-w.

## Introduction

Over a century ago, Ramón y Cajal identified a distinct population of gut cells with unique features and named them interstitial neurons. With the increased use of transmission electron microscopy (TEM) in the 1970s, researchers observed a significant difference in the ultrastructure of these cells compared with typical neurons. Consequently, the name was revised to Cajal’s interstitial cells (ICC) to reflect their distinct morphology. However, further investigation revealed a unique combination of morphological and immunophenotypic features not previously recognized in other cell types. This led to the designation of interstitial Cajal-like cells (ICLC) in recognition of their resemblance to Cajal’s original description but with distinct properties. Finally, to emphasize their unique identity, the term telocyte (TC) was adopted for these cells (Purelku and Tanriverdi 2023)**.**

TCs are one of the relatively new identified untypical interstitial cell types with exceptionally long and thin prolongations extended from their cell bodies, known as telopodes (Tps) (Cretoiu and Popescu 2014a). Since the first stages of their characterisation, the “gold standard” for their identification has been TEM (Popescu et al. 2007). Ultrastructurally, TCs are characterized by a small oval-shaped cell body (9–15 μm) and a variable number (one to five) of Tps, with alternating regions of podomers (Pdms) (∼80 nm) and podoms (Pds) (250–300 nm). Their presence is identified in a wide range of species such as reptiles, fish, birds and mammals, including humans, in both cavitary and non-cavitary organs (Cretoiu and Popescu 2014a). All of the cells identified as TCs were found to be widely prevalent within the connective tissue and organized in a three-dimensional (3D) network, dispersed in the extracellular matrix and establishing stromal synapses (Popescu et al. 2005b). These communications may occur with resident cells, such as fibroblasts, mast cells and adipocytes, as well as with non-resident cells, such as immune cells from the bloodstream, or at the connective boundary of various tissues (e.g., epithelial, muscular and nerve tissues) lining and surrounding blood vessels. TCs found near blood vessel walls are considered to function as adventitial cells (Pellegrini and Popescu 2011). TCs may be integrally engaged in the maintenance of tissue homeostasis and regeneration via short- and long-distance intercellular communication (Cretoiu and Popescu 2014b). They are found in tissues where cells are sparsely distributed, with substantial gaps between them, indicating that Tps might aid in connecting cells over extended distances (Hatta et al. 2012). They also have a strategic position in regard to stem cell niches (Popescu et al. 2009a, 2011; Gherghiceanu and Popescu 2010; Luesma et al. 2013) making them ideal candidates for long-distance cell-to-cell communication, immunological surveillance (Carmona et al. 2011) and stem cell niche supervision (Popescu et al. 2009a; Luesma et al. 2013).

In parallel, several immunohistochemical markers including CD117/c-kit, vimentin (Cantarero et al. 2015; Klein et al. 2022a, b), desmin, α-SMA, progesterone receptor, estrogen receptor, S100 protein, CD34 and PDGFR-β have been employed for TC detection (Janas et al. 2018). Nevertheless, there is no specific marker that fully characterizes the TCs in order to distinguish them from the other interstitial cells. For that reason, the morphology of these cells is crucial for identifying them under light microscopy. As a result, due to lack of a specific marker TEM remains a key technique for identifying TCs in different tissues and organs (Roatesi et al. 2015).

TCs are present in practically all mammalian tissues such as the gastrointestinal (Popescu et al. 2005c; Hinescu et al. 2007; Carmona et al. 2011), cardiovascular (Popescu et al. 2009b; Zhang 2016), respiratory (Zheng et al. 2011), urinary (Gevaert et al. 2012; Qi et al. 2012) and reproductive (Popescu et al. 2005a; Hatta et al. 2012) systems and they have been investigated to varying degrees in the majority of the systems mentioned, with some being examined slightly and others being studied more extensively. The female reproductive system’s organs fit into the latter group (Klein et al. 2020); however, they must be researched in more depth and given more serious consideration.

TCs in the female reproductive system have been discovered in the uterus, uterine tubes (Popescu et al. 2007), vagina (Shafik et al. 2005), mammary gland (Gherghiceanu and Popescu 2005) and placenta (Nizyaeva et al. 2017), as well as in the ovaries (Liu et al. 2016; Mazzoni et al. 2019) of various species. Although TCs are relatively new identified interstitial cells, pathologies that have relationship with TCs have gained the “telocytopathies” term pointing out different diseases including the ones in the female reproductive system. Tubal TCs, which are located near smooth muscles, have been impacted during various pathological changes in the uterine tube thereby affecting tubal motility, and TCs in the uterus have also been linked to both idiopathic and explainable infertility, as well as associated with leiomyomas or fibroids (Roatesi et al. 2015; Skowron et al. 2018; Klein et al. 2022a, b). However, interestingly, it also becomes apparent that research on specific organs in rats which are widely used to model human physio(patho)logy is relatively limited, particularly when it comes to the female reproductive organs. In that context, not all organs in the female reproductive system of rats, such as the ovaries, have been investigated for the presence of TCs. Therefore, this article aimed to demonstrate the existence of TCs and their histomorphology along with their ultrastructural properties in the rat ovaries for the first time, as well as give more detailed insight of the previously documented TCs in rat uteri.

## Methods

### Experimental animals and tissue processing

This study received approval from the Bezmialem Foundation University Laboratory Animals Local Ethics Committee under the reference number 31.10.2023-E.127867. Young adult Sprague–Dawley female rats (*n* = 8) were maintained in an environment with a constant room temperature, following a 12-h light and dark cycle, and provided with ad libitum access to standard laboratory chow and water. Estrous cycles of the rats were observed for a duration of 7 days using vaginal smear method to check the general reproductive health of the rats. Rats with regular estrous cycle were considered to be included in the study. Subsequently, ovaries and uteri were extracted from the animals that were under anesthesia. Approximately 1 mm^3^ tissues from the left ovaries and uteri were preserved in 2.5% glutaraldehyde while the right ovaries and uteri underwent fixation with 4% formaldehyde overnight. The right ovaries and right uteri were furthermore dehydrated through a ascending ethanol series, with concentrations of 70°, 80°, 90°, 96° and 100°, respectively for further analysis. Following dehydration, clearing was performed using toluene. Subsequently, paraffin wax was utilized for embedding and histological sections of 4 µm were obtained.

### Transmission electron microscopy

First of all, the left ovaries and left uteri were post-fixated using a 1% OsO_4_ solution. Then tissues were washed with phosphate buffer. Subsequently, they were dehydrated through a ascending ethanol series, with the concentrations of 50°, 70°, 80°, 90°, 96° and 100°, respectively. Following dehydration, they were treated with mix of propylene araldyte solutions in ratios of 3:1, 1:1 and 1:3, respectively. Afterwards, the tissues were embedded in araldyte, and underwent a 2-day polymerization process at 60 °C. Finally, semi-thin sections of approximately 1 µm were obtained and stained with toluidine blue, while ultrathin sections of 50–60 nm were acquired using ultramicrotome (Reichert UM3). The thin sections were placed on copper grids and subjected to contrast staining with uranyl acetate and lead citrate solutions. The grids were observed under a transmission electron microscopy (TEM) (JEOL, JEM-1011, Japan). The size of TCs were measured with ImageJ software using line tools (Schneider et al. 2012).

### Immunohistochemistry

Following deparaffinization and rehydration, antigen retrieval using citrate buffer (pH 6) was executed. Washing with phosphate-buffered saline (PBS) was conducted after each successive step. H_2_O_2_ and serum blocking steps were then performed. The tissues were subsequently incubated with either rabbit anti-c-kit (1:100 dilution, STJ92298, St. John’s Laboratory, UK) or rabbit anti-PDGFR-β (1:200 dilution, STJ95003, St. John’s Laboratory, UK) primary antibodies overnight at 4 °C. Following this, a broad spectrum horse radish peroxidase (HRP) secondary antibody kit (HRP060, HRP-S-500, Zytomed, Germany) was employed, with 3,3′-diaminobenzidine (DAB) serving as the chromogen. A negative control was performed without incubation of the primary antibodies to determine non-specific staining (Figure S1).

Five random areas of ovarian stroma and endometrial stroma of the uterus were imaged with ×200 magnification by using bright-field microscope (Olympus, BX61, Japan). Next, a quantification analysis was conducted on cells expressing either c-kit or PDGFR-β, while also manifesting a telocyte-like cell (TCLC) morphology (Iancu et al. 2018; Petrea et al. 2018). The objective was to assess whether the counts of TCLCs differed and whether they exhibited comparability by each singular antibody marker. The purpose of this assessment was to ascertain the concordance in the numbers of TCLCs designated by a single antibody, shedding light on potential correlations between these markers and the identified TCLCs phenotype. Single immunopositive cells (c-kit or PDGFR-β ), when considered alongside their morphology, were termed as TCLC rather than TC. The same terminology, TCLC, was also applied to semi-thin sections.

For double labelling a combined method, utilizing both immunoperoxidase and immunofluorescence (Lechago et al. 1979) was employed to demonstrate TCs with two different markers. The rationale behind this methodological choice is the need for more precise identification of TCs through immunohistochemistry, which often requires double-labelling techniques for accurate characterization. The selection of immunoperoxidase aimed at facilitating the identification of TC morphology, while immunofluorescence was employed for the purpose of double labelling, contributing to a comprehensive understanding of TC morphology and marker expression. Thus, following deparaffinization and rehydration, the heat-induced antigen retrieval method was applied with citrate buffer (pH 6) using a microwave for 20 min. PBS was used to wash the tissues after each step. After the H_2_O_2_ and serum blocking steps, the tissues were incubated with an rabbit anti-α-SMA primary antibody (dilution of 1:300, Cat. No. E-AB-34268, Elabscience, TX, USA) overnight at 4 °C. The next day, tissues were washed away from the primary antibody and goat-raised anti-rabbit IgG secondary antibody (Alexa flour 568) (cat. no. ab175471, Abcam, UK) was used. After that, mouse anti-CD34 primary antibody (dilution of 1:10, Cat. No. orb388911, Biorbyt, MO, USA) was incubated with the tissues overnight at 4 °C. Following that, a broad spectrum HRP secondary antibody kit (HRP060, HRP-S-500, Zytomed, Germany) was employed, with 3,3′-diaminobenzidine (DAB) chromogen. Additionally, 4 6-diamidino-2-phenylindole (DAPI) was employed as a counterstaining agent. A negative control was performed without incubation of the primary antibodies to determine non-specific staining (Figure S1).

Ovarian stroma and endometrial stroma of the uterus were imaged at ×400 magnification using a microscope that supports both bright-field and fluorescent imaging (Olympus, BX61, Japan). Subsequently, the region of interest was photographed first in bright-field to visualize the CD34+ areas. Then, the same region was photographed in appropriate fluorescent channels to show α-SMA+ and DAPI+ areas. The images were then merged to demonstrate the double-stained areas for CD34 and α-SMA. The identification of TCs was performed based on either the double-stained of CD34 and α-SMA cell body of TCs, which also shows DAPI+ nuclei, or their double-stained Tps with CD34 and α-SMA, which are thin, elongated structures.

### Statistical analysis

Data were analyzed using IBM SPSS 29.0 (IBM Corp., Armonk, NY). An independent *t*-test was run on the ovaries and uteri to compare between numbers of c-kit+ and PDGFR-β+ TCLCs. *p* < 0.05 is accepted as statistically significant and the results are presented as the mean (M) ± standard error of mean (SEM).

## Results

### TCs are distributed throughout the rat's ovarian stroma and strategically localized in specific areas

**Fig. 1:**
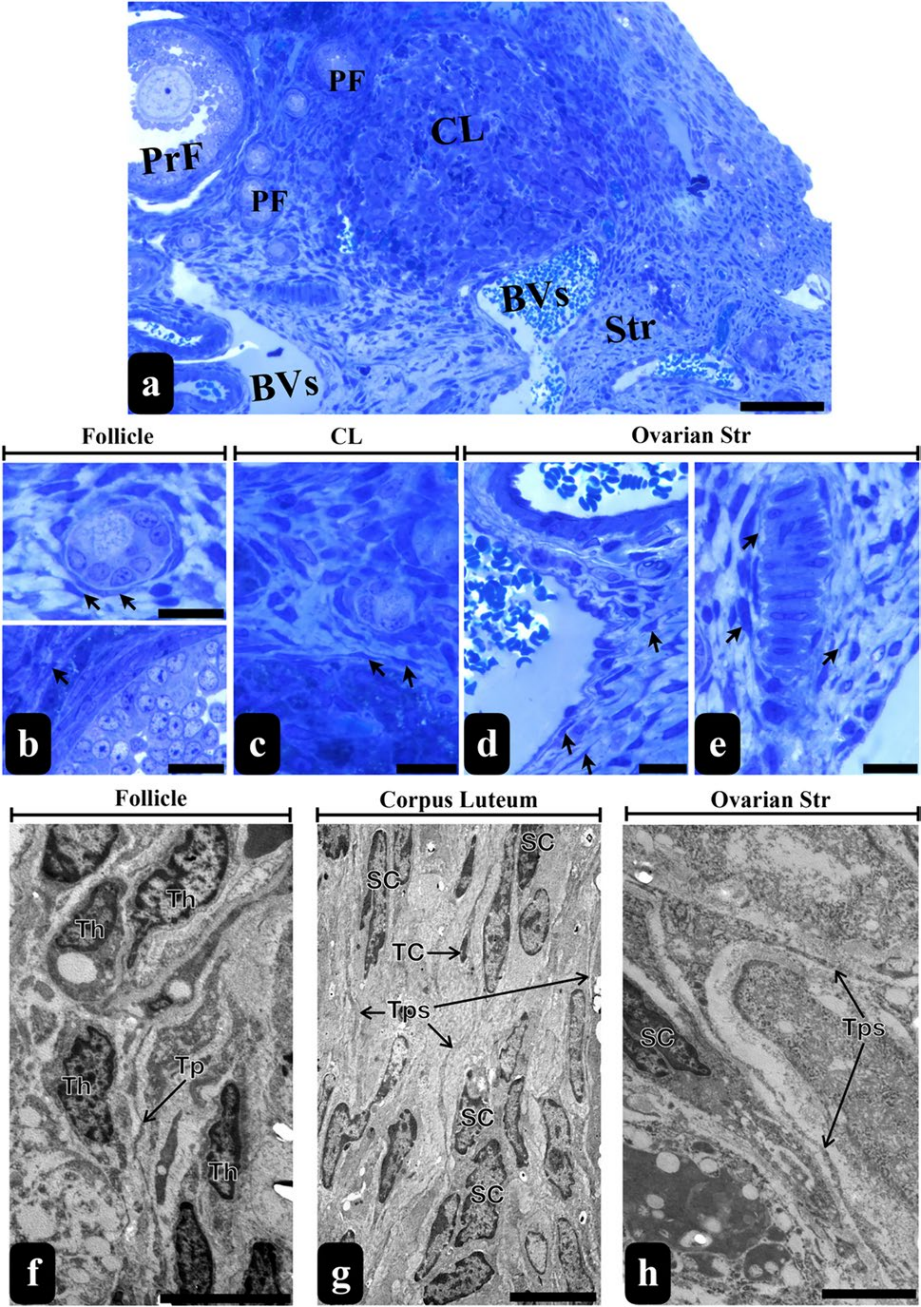
Semi-thin and thin sections of rat ovaries. The micrograph stained with toluidine blue show semi-thin section from the ovarian cortex and medulla (scale bar, 50 µm) (**a**). These regions consist of a number of follicles in different developmental stages (**b**) and corpus luteum (**c**), as well as blood vessels (**d**) and nerve endings in the stroma (scale bar, 10 µm) (**e**). The arrows in the micrographs (**a**–**e**) indicate the TCLCs. The thin sections exhibit Tps surrounding the follicles more specifically in between the thecal layers (scale bar, 5 µm) (**f**), around the corpus luteum (scale bar, 5 µm) (**g**) and throughout the ovarian stroma (scale bar, 2 µm) (**h**). *PrF* primary follicle, *PF*  primordial follicle, *BV* blood vessel, *CL* corpus luteum, *Str* stroma, *Th* theca cells, *TC* telocyte, *Tp* telopods, *SC* stromal cell

Upon examination of toluidine blue-stained semi-thin sections of rat ovaries, TCLCs were noted to exhibit a distribution surrounding follicles at various developmental stages (Fig. 1b), the corpus luteum (Fig. 1c) and extensively within the ovarian stroma (Fig. 1d, e). Notably, TCLCs were prominently situated around the blood vessels (Fig. 1d), also around some nerve endings (Fig. 1e) accompanied by their Tps.

**Fig. 2:**
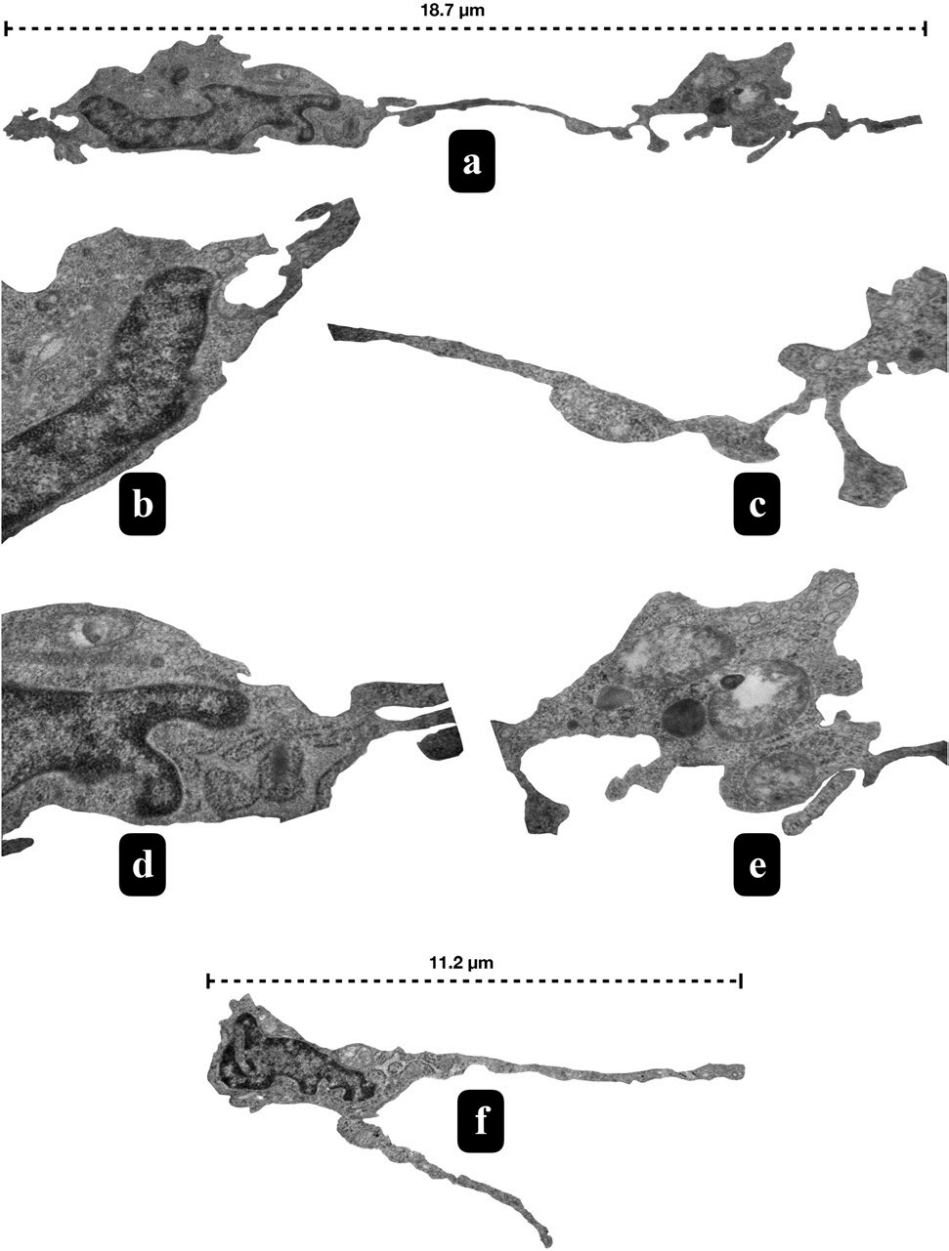
Comparative ultrastructure of the TCs of rat ovary and uterus. Ovarian TCs were measured up to 18.7 µm in length together with their cell body (**a**). Their cell body showed pleomorphic shape while their nuclei revealed peripheral heterochromatic and central euchromatic areas (**a**, **b** and **d**). They showed prolongations as Tps with a thinner segments called as Pdms (**a**, **c**) and dilated segments enriched with organelles (**a**, **e**). Uterine TCs were measured up to 11.2 µm in length together with their cell body sharing a similar ultrastructure with ovarian TCs (**f**)

**Fig. 3:**
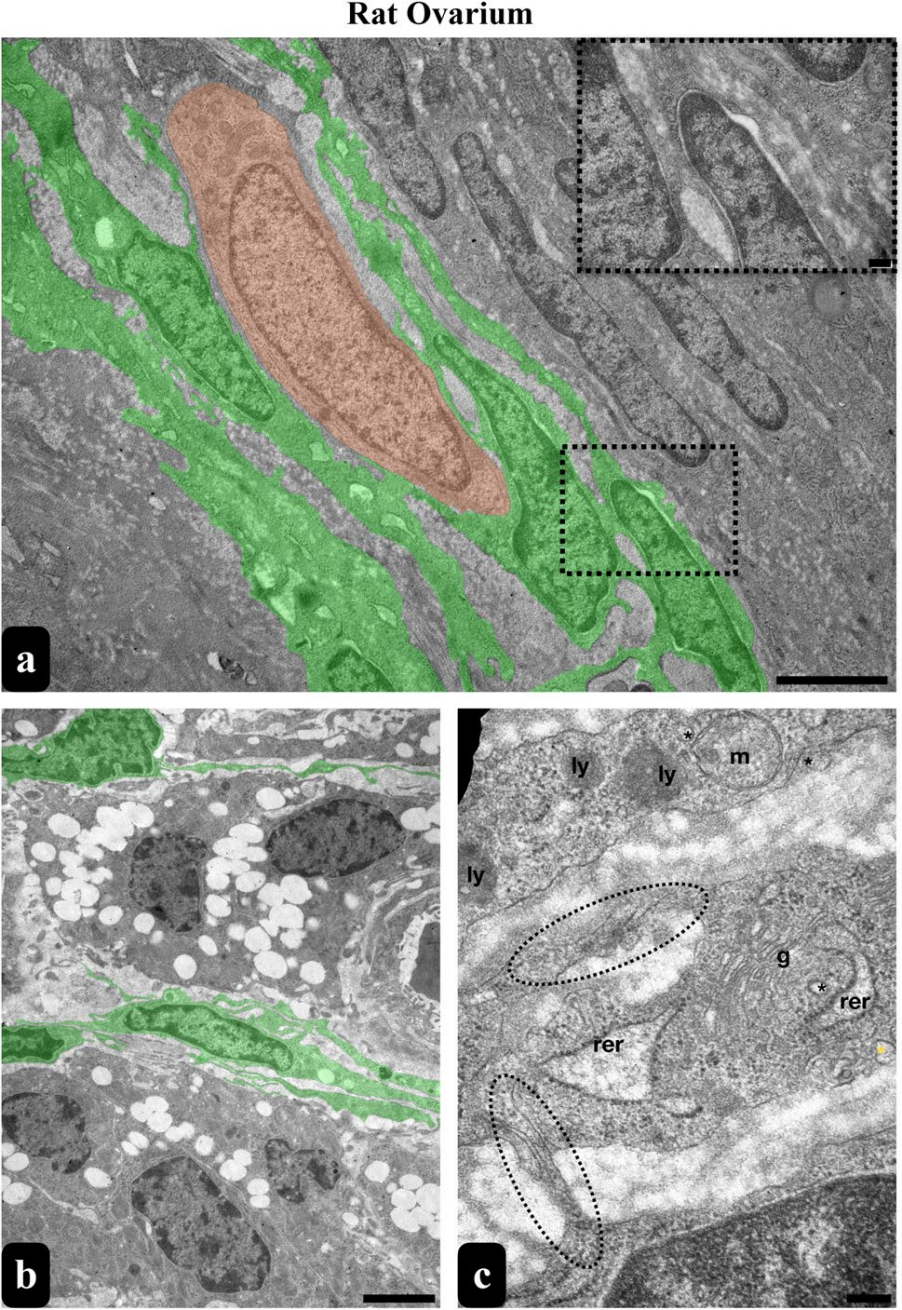
TEM micrograph of rat ovarian stroma exhibit TCs and Tps (coloured in green) forming close contacts with each other (inset) as well as other stromal cells such as fibroblasts coloured in orange (Scale bar: 2 µm, Inset’s Scale Bar: 200 nm) (**a**). TCs and Tps among the connective tissue of the corpus luteum were coloured in green (scale bar, 2 µm) (**b**). A higher magnification of Tps indicates the presence of organelles such as RER, Golgi, mitochondria, lysosomes, caveolae and vesicles as well as inter-digitations with other Tps (scale bar, 200 nm) (**c**). *ly* lysosome, *m* mithocondria, *g* golgi, *rer* rough endoplasmic reticulum, black * represents vesicles, yellow * represents caveolae, dashed elipses represent inter-digitations

Subsequent TEM analysis of these designated regions of interest substantiated the identity of these cells as TCs. TCs demonstrated a distinctive profile, characterized by pleomorphic cell bodies and nuclei featuring peripheral heterochromatin and central euchromatin (Figs. 1g and 2a, b, d). Their cell bodies contained minimal cytoplasm, whereas their remarkably elongated cytoplasmic extensions, termed Tps were densely populated with organelles (Figs. 1f, g, h, 2 and 3). Noteworthy components within the Tps included thin segments known as Pds (Figs. 2 and 3) and their dilated segments referred to as Pdms (Figs. 2e and 3c). Within these extensions, particularly in the Pdms, an abundance of rough endoplasmic reticulum (RER), Golgi apparatus, ribosomes, endocytotic vesicles, lysosomes and autophagic figures were observed. These distinctive cells exhibited close associations or connections with other cell types, as well as interactions among themselves (Figs. 1 and 3a). The dimensions of the rat ovarian TCs were measured to be up to 18.7 µm (Fig. 2a).

Ultrastructural analysis provided a detailed localization of the position of TCs. Our findings indicate that TCs, along with their Tps, are situated within the theca layers of the follicles, specifically in the connective tissue between these layers (Fig. 1f); however, they do not establish contact with the granulosa cells. Conversely, TCs with Tps and Pdms were observed surrounding the corpus luteum, in proximity to luteinized granulosa cells, and coexisting with other interstitial cells in the connective tissue (Fig. 1g). In contrast, numerous TCs dispersed in the stroma exhibited dichotomous or trichotomous branching with their Tps, facilitating connections with Tps of adjacent TCs and other cells (Figs. 1h and 3a–c). Furthermore, TCs within the stroma appeared to be habitually positioned around blood vessels, particularly in close proximity to pericytes and smooth muscle cells (Fig. 3b).

### The meshwork of TCs in the rat uterine appears to be associated with various cell types

**Fig. 4:**
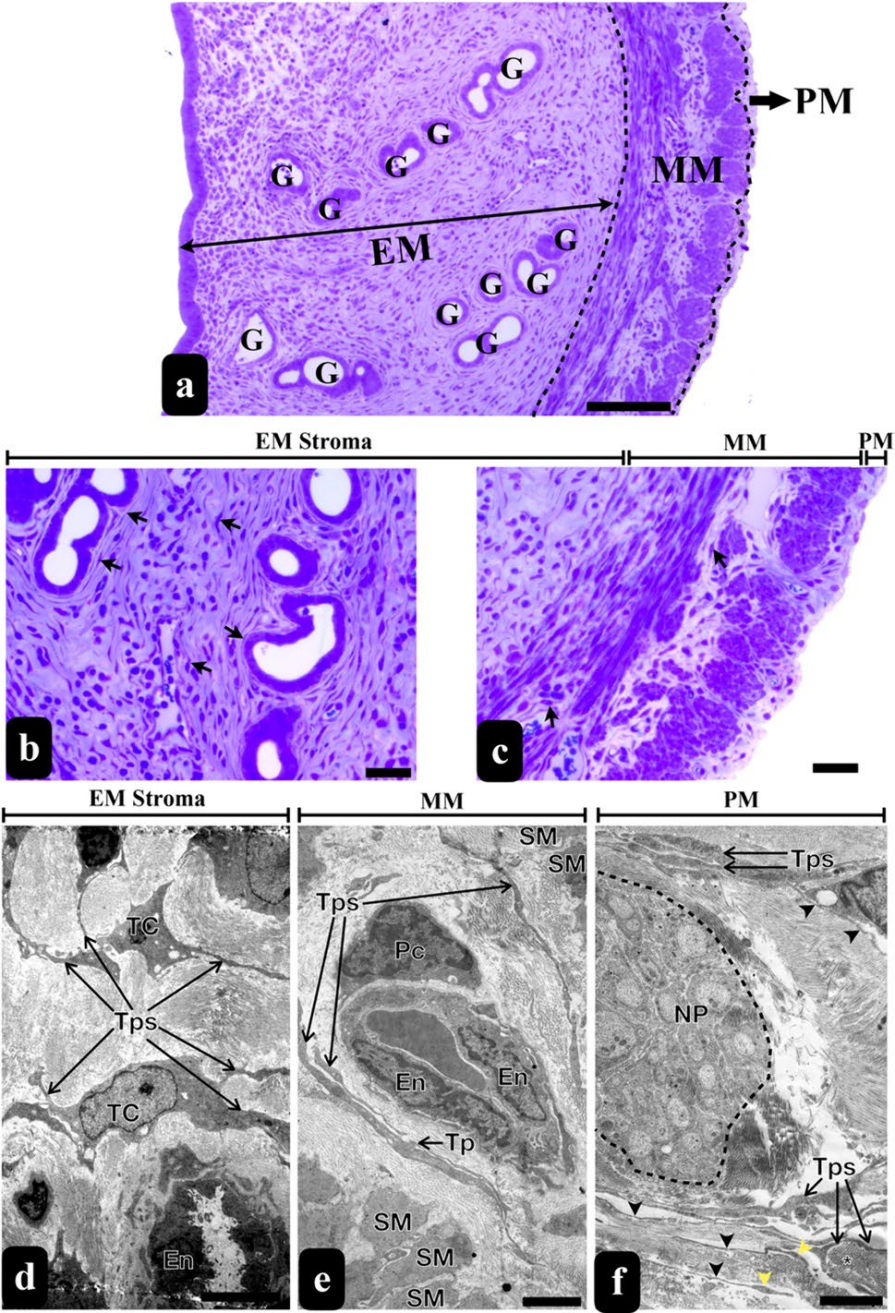
Semi-thin and thin sections of rat uteri. The micrographs stained with toluidine blue show semi-thin sections from the uterus. These sections demonstrate the endometrial, myometrial, and perimetrial layers of the uterus (scale bar, 50 um) (**a**). The arrows in the micrographs (**b** and **c**) indicate the TCLCs. TCs were seen within the endometrial stroma surrounding the uterine glands (**b**), between the muscle layers of the myometrium and surrounding the blood vessels and nerve packages of the perimetrium (**c**) uterus (scale bar, 20 um). The thin sections exhibit TCs with their Tps along with their Pds and Pdms through the endometrial stroma (scale bar, 5 um) (**d**), between the smooth muscle cells surrounding capillaries in the myometrium (scale bar, 2 um) (**e**), and surrounding nerve packages in the perimetrium (black arrowheads: podomer of Tps, yellow arrowheads: podome of Tpsi, * represents collagen fibers (scale bar, 2 um) (**f**). *EM* endometrium, *MM* myometrium, *PM* perimetrium, *G* gland, *TC* telocyte, *Tp* telopod, *En* endothelial cell, *Pc* pericytes, *SM* smooth muscle, *NP* nerve package

Analyzing semi-thin sections stained with toluidine blue from the rat uterus uncovered the existence of TCLCs in all three layers: the endometrium, myometrium and perimetrium. Closer examination of TCLCs in the endometrial stroma revealed that the Tps were not solely restricted to surrounding blood vessels; they were also identified around the uterine glands (Fig. 4a–c).

**Fig. 5:**
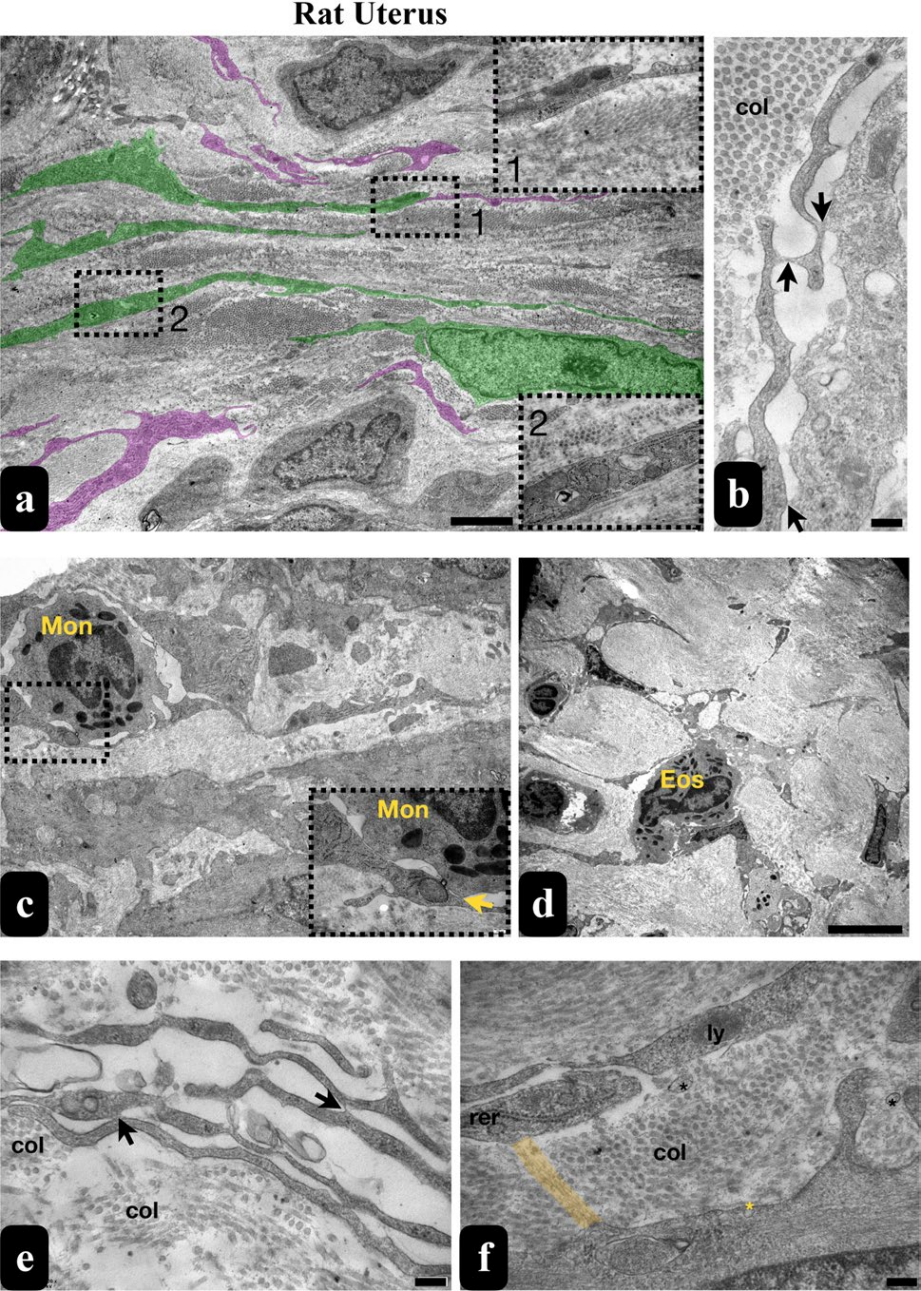
TEM micrographs of rat uterine stroma exhibit TCs and Tps (coloured in green and purple) while inset 1 indicates the contact of Tps with each other, inset 2 shows a Pd enriched with organelles which are the higher magnifications of the selected areas (scale bar, 2 μm) (**a**). Homocellular connections of Tps among themselves were observed (Scale bar: 200 nm) (**b**, **e**). TCs in the stroma formed heterocellular connections with monocytes (**c** and inset) and eosinophils (scale bar, 5 μm) (**d**). Long and thin protrusions of the cytoskeletal structures derived from a stromal cell to a Tp were also detected (yellow coloured shade) (scale bar, 200 nm) (**f**). Mon monocyte, *Eos* eosinophil, *ly* lysosome, *rer* rough endoplasmic reticulum, *col* collagen fibers, black * represents vesicles, yellow * represents caveolae, black arrows represent homocellular connections, yellow arrows represent heterocellular connections

The ultrastructural characteristics of uterine TCs closely resembled to the ovarian TCs. In the uterine stroma, TCs were interspersed among other resident interstitial cells such as fibroblasts and immune cells such as monocytes and eosinophils (Fig. 5a, c, d), suggesting a potential involvement in immunological processes in the rat uterus. TCs were also identified in the connective tissue between the smooth muscle layers of the myometrium (Fig. 4e) and perimetrium (Fig. 4f), analogous to the endometrial stroma. Their close relationships with nerve endings were also noted (Fig. 4f). These TCs exhibited diverse hetero and homocellular connections, with their Tps forming inter-digitations and potentially facilitating the intercommunication (Fig. 5a, b, e, f). Suprisingly, we managed to detect long and thin protrusions of the cytoskeletal structures derived from a stromal cell to a Tp (Fig. 5f). The dimensions of the rat uterine TCs were measured up to 11.2 µm (Fig. 2f).

### While double labelling confirms the TCs of ovary, immunohistochemistry reveals a correlation between the quantification of c-kit+ TCLCs and PDGFR-β+ TCLCs in both the ovary and uterus

Following our ultrastructural findings, our objective was to demonstrate TCs with two different well identified markers by using double labelling with CD34 and α-SMA for TCs in the stroma of rat ovary and the endometrium of rat uterus. We also separately utilized c-kit and PDGFR-β for semi-quantitatively assessment for the presence of TCLCs throughout the both organs.

**Fig. 6:**
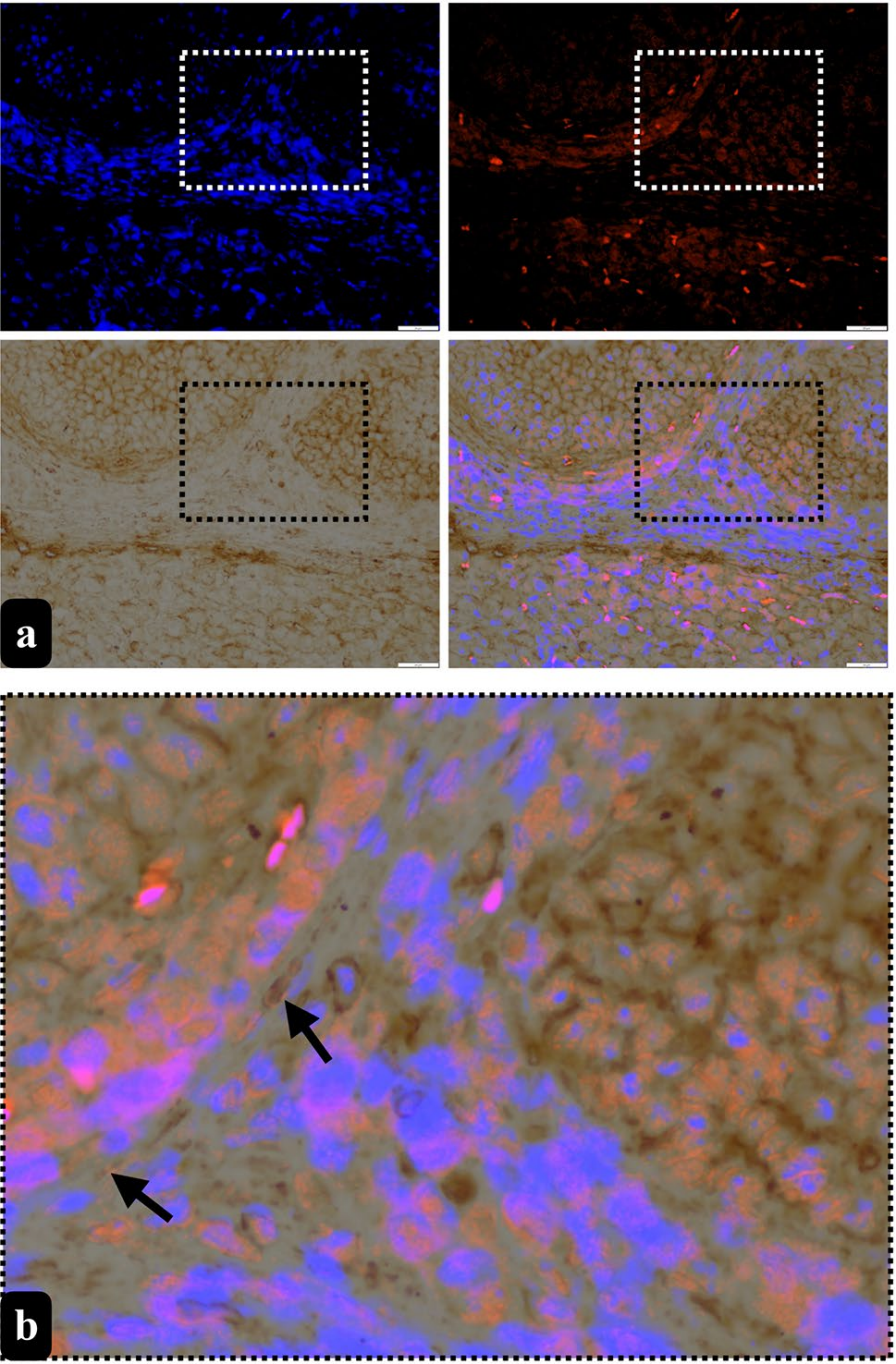
Double-labelled TCs (arrow) with α-SMA (red, marked with fluorescent dye) and CD34 (brown, marked with DAB) counterstained with blue to show the nuclei (DAPI) were presented in lower (**a**) and higher (**b**) magnification in the stroma of rat ovaries (scale bar, 20 µm)

**Fig. 7:**
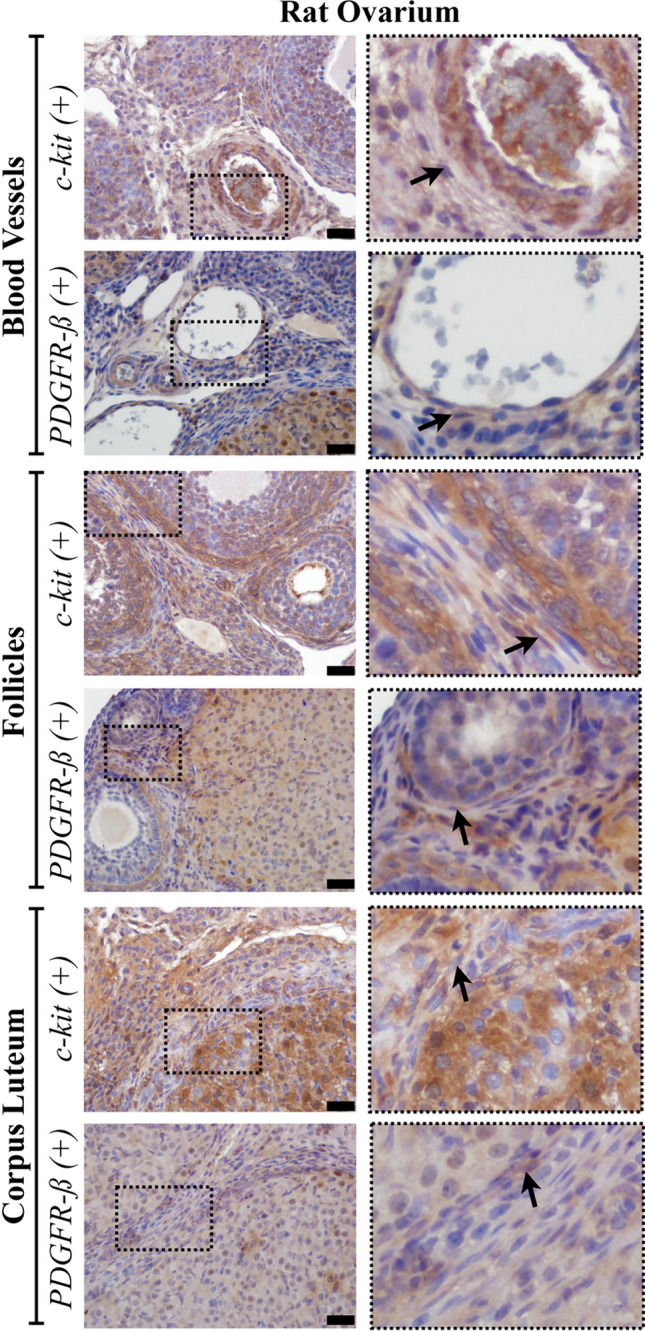
Micrographs of singled-labelled c-kit or PDGFR-β-positive cells for semi-quantitatively assessment for the presence of TCLCs throughout the rat ovarian stroma. Arrows indicate TCLCs (scale bar, 20 µm)

**Fig. 8:**
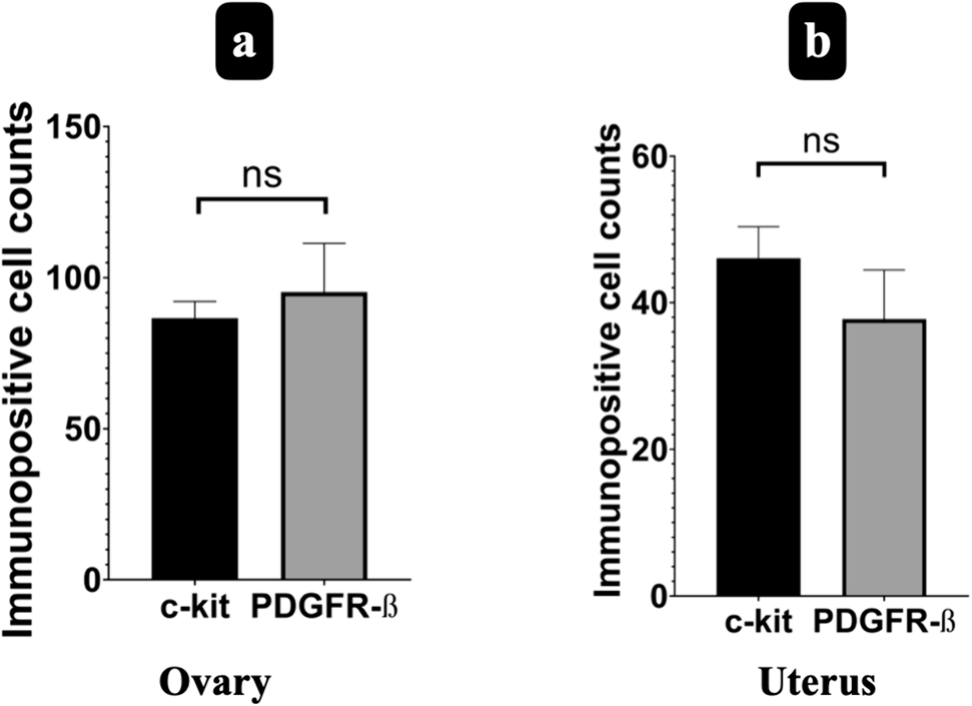
Comparison for number of PDGFR-β+ TCLCs and c-kit+ TCLCs in the rat ovarian stroma (**a**) and endometrial stroma of uterus (**b**). There was no statistical significance (ns) between the PDGFR-β+ and c-kit+ cells for both organs (*p* > 0.05)

Double-labelled TCs with α-SMA and CD34 and single-labelled TLCLs with c-kit+ or PDGFR-β+ were evident in the stroma of rat ovaries, particularly in proximity to blood vessels, follicles and the corpus luteum (Figs. 6 and 7). PDGFR-β+ TCLCs were counted as 95.24 ± 16.13 in ovarium stroma while c-kit+ TCLCs were counted as 86.6 ± 5.52. When an independent *t*-test was run on the data with a 95% confidence interval (CI) for the mean difference between the counted PDGFR-β+ and c-kit+ TCLCs for the ovarian stroma (*t* (8) = 0.507, *p* = 0.137), there was no statistical significance between the counted cells (Fig. 8a).

**Fig. 9:**
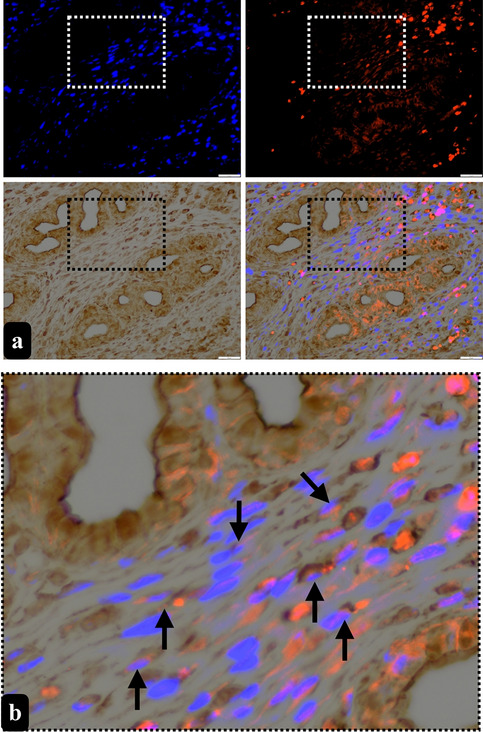
Double-labelled TCs (arrow) with α-SMA (red, marked with fluorescent dye) and CD34 (brown, marked with DAB) counterstained with blue to show the nuclei (DAPI) were presented in lower (**a**) and higher magnification (**b**) in the stroma of rat endometrium (scale bar, 20 µm)

**Fig. 10:**
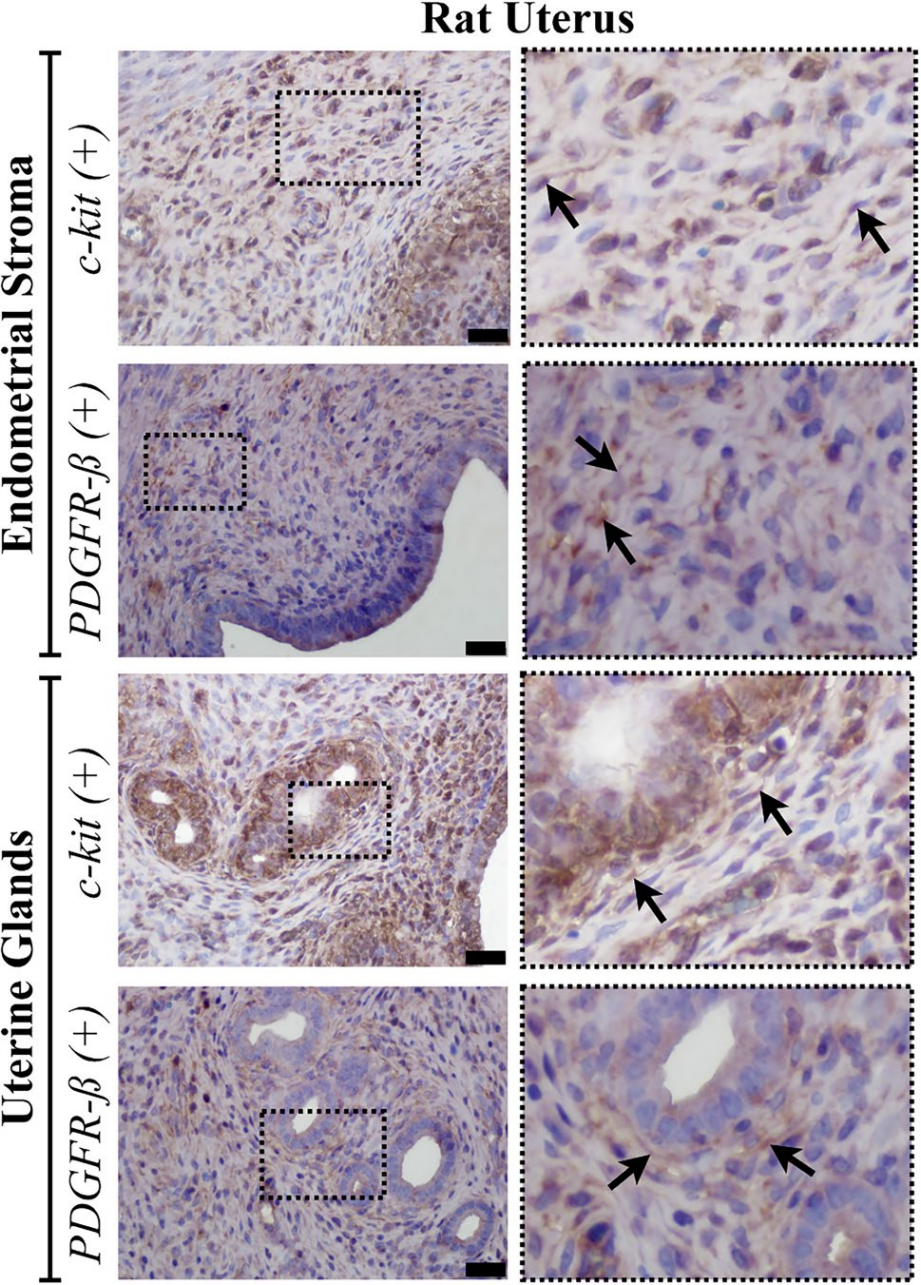
Micrographs of single-labelled c-kit or PDGFR-β positive cells for semi-quantitatively assessment for the presence of TCLCs throughout the rat endometrial stroma. Arrows indicate TCLCs (scale bar, 20 µm)

Conversely, within the uterus, double-labelled TCs with α-SMA and CD34 and single-labelled TLCLs with c-kit+ or PDGFR-β+ were observed in the endometrium, myometrium, and perimetrium. In the endometrium, TCLCs were predominantly situated near blood vessels and uterine glands, although their presence extended throughout the stroma. Subsequently, TCLCs were identified between the muscle layers of the myometrium and specifically within the perimetrial stroma, particularly surrounding blood vessels and nerve bundles (Figs. 9 and 10). All these immunohistochemical observations align with and support our semi-thin/ultrastructural analysis. PDGFR-β+ TCLCs were counted as 37.76 ± 6.72 in endometrial stroma of the uterus while c-kit+ TCLCs were counted as 46.08 ± 0.6. When an independent *t*-test was run on the data with a 95% confidence interval (CI) for the mean difference between the counted PDGFR-β+ and c-kit+ TCLCs for the endometrial stroma (*t* (8) = −1.042, *p* = 0.450), there was no statistical significance between the counted cells (Fig. 8b).

## Discussion

Ovarian TCs represent a relatively understudied aspect within the female reproductive system, with no prior investigation reported in rat ovaries to the best of our knowledge. A study conducted by Liu et al. (2016) explored TCs in the ovarian stroma of mice, utilizing electron microscopy, immunofluorescence, immunohistochemistry and flow cytometry by employing some markers such as CD34, vimentin and PDGFR-α and -β. The researchers found these cells characterized by a small body and a moniliform transitioning arrangement of branching Tps. Furthermore, it was suggested that TCs may play a role in sustaining the local microenvironment.

In our study, we have successfully demonstrated, for the first time, the presence of TCs in rat ovaries. Moreover, our investigation revealed a widespread distribution of TCs throughout the ovarian tissue. The observed morphology of TCs in the ovary closely resembled that reported in various other studies (Liu et al. 2016; Mazzoni et al. 2019; Mohamedien et al. 2023). Importantly, we observed an abundance of Tps and Pdms throughout the ovarian stroma, indicating a close interrelation among these structures and with other stromal cells. This supports the notion that TCs may form intricate three-dimensional networks via homo- and heterocellular connections, as previously proposed by Cretoiu and Popescu (2014b). Notably, our findings also align with existing data, as TCs in the rat ovary exhibited Tps densely populated with organelles, including RER, caveolae, Golgi apparatus, ribosomes, endocytic vesicles, lysosomes and autophagic figures. The presence of these organelles within the Tps underscores the high metabolic activity of these cells (Lemons et al. 2010; Felisbino et al. 2019). Furthermore, consistent with prior knowledge, Tps are recognized for their ability to secrete three forms of extracellular vesicles which are exosomes, ectosomes and multi-vesicular cargos implicated in paracrine signalling (Cretoiu and Popescu 2014b, a). In this study, we report that the nuclei of TCs in both the ovary and uterus consist of centrally euchromatic regions with peripheral heterochromatin. However, in various studies, there is a debate regarding the dominant presence of heterochromatin in the nuclei of TCs. Similar to our findings, some researchers propose that TCs exhibit nuclei with irregular indentations, central euchromatin and heterochromatin concentrated near the nuclear membrane. Studies also assert that TCs typically feature nuclei characterized by thin heterochromatin band and infrequently observable nucleoli (Cretoiu et al. 2012; Cretoiu and Popescu 2014b, a; Condrat et al. 2021).

Studies on TCs in fish ovaries have elucidated their role in establishing intricate connections between stromal and vascular components, forming a labyrinth network. These TCs have been shown to engage with immune cells and blood vessel endothelium, shedding extracellular vesicles in close proximity to blood capillaries, and contributing to tissue regeneration during the spawning season (Mokhtar 2019; Mohamedien et al. 2023). Additionally, research on rabbit ovaries has proposed a potential involvement of TCs in the formation and regeneration of theca interna, interstitial gland cells and theca lutein cells (Abd-Elkareem 2017).

Consistent with these findings in various species, our study observed the presence of TCs, along with their Tps, situated between the connective tissue of the theca layers. Interestingly, these TCs did not establish direct contact with granulosa cells. In contrast, TCs with their Tps and Pdms were specifically localized around the corpus luteum, in close proximity to luteinized granulosa cells as well as with other interstitial cells. Moreover, TCs within the stroma exhibited a consistent positioning around blood vessels, particularly in close proximity to pericytes and smooth muscle cells, aligning with the observed patterns in previous studies across different species as mentioned in other studies (Mokhtar 2019; Mohamedien et al. 2023).

On the contrary, our examination of uterine TCs revealed their presence in all three layers, namely the endometrium, myometrium and perimetrium. A study conducted by Salama (2013) has already demonstrated the existence of TCs in the endometrium and myometrium of the rat uterus across various reproductive stages. The suggested roles of these TCs include providing support to glands and facilitating stromal cell communication in the endometrium. In the myometrium, their proposed functions involve initiating and coordinating myometrial contractions.

In parallel with the observations in the ovaries, our investigation reveals that Tps and Pdms form an extensive meshwork throughout the stroma of all three layers of the uterus. Tps in the rat uterus exhibit variable distances when connecting to other cells, suggesting a range of cell-to-cell communication. Additionally, Tps are known to establish connections with each other through end-to-end or side-to-side contacts, but rarely through end-to-side connections in the uterus. These morphological observations provide evidence supporting the notion that Tps may not only convey signals or engage in unique communication with other TCs but also with neighbouring stromal cells (Hatta et al. 2012; Yang et al. 2016). The protrusions of the cytoskeleton driven from a stromal cell to a Tp seem to be tunnelling nanotubes or cytonemes. These types of communications are known to allow the transfer of different cargoes, ranging from ions to organelles (Abounit and Zurzolo 2012; Gerdes et al. 2013; Korenkova et al. 2020). We also noted that the Tps, especially their Pdms in the uterine tissue were abundant in organelles, displaying a similar feature observed in TCs in the rat ovary.

Furthermore, existing literature reports the presence of Tps in the uterus, positioned in close proximity to secretory glands and blood vessels in the lamina propria beneath the simple columnar epithelium. Notably, close connections between TCs and smooth muscle fibres, facilitated by gap junctions and connective tissues, have been observed (Yang et al. 2016). This aligns with observations in the human endometrial stroma, where TCs are present in both the stratum functionalis and basalis, adopting an orientation that contours the shape of the adjacent epithelial architecture (Hatta et al. 2012).

Aligned with these coherent observations, our study reveals that Tps are not confined solely to the vicinity of blood vessels; they were also identified around uterine glands. TCs were further identified in the connective tissue between the smooth muscle layers of the myometrium and in the perimetrium, mirroring the distribution observed in the endometrial stroma. Previous research has proposed that TCs could exert influence on the timing of contractile activity in smooth muscle cells, highlighting their potential key role in uterine contraction (Yang et al. 2016). In connection with this, there is a suggestion that TCs may contribute to a range of physiological functions in both non-pregnant and pregnant states of the human uterus, which is recognized for its myogenic contractions (Roatesi et al. 2015).

We found that TCs were distributed among other resident interstitial cells, including mast cells, indicating a potential involvement in immunological processes in the rat uterus. Jiang et al (2018) investigated the immunomodulatory capabilities of uterine TCs, particularly their influence on macrophages. This study revealed that TCs could directly activate these innate immune cells through the mitochondrial signalling pathway. Although the experiment was conducted on peritoneal macrophages, the findings suggest that similar processes may occur in the uterine microenvironment.

It is known that TC markers such as c-kit and PDGFR-ß (Suciu et al. 2012; Salama 2013; ES 2022), which are used in this study, can also be expressed by other cell types found in connective tissue. Despite some researchers using one of these antibodies to identify TCs based on their morphology, the double staining method is considered more reliable and is commonly employed. Therefore, we used c-kit and PDGFR-β antibodies to identify TCLCs based on their morphological characteristics. Our next objective was to assess semi-quantitatively the presence of TCLCs in both the stroma of the rat ovary and in the endometrium of the rat uterus. This assessment helped us to ascertain the concordance in the quantitiy of TCLCs designated by a single antibody, shedding light on potential correlations between these markers and the previously identified TCLCs phenotype. The non-significant results between the c-kit+ and PDGFR-β+ TCLCs support the conclusion that a significant number of the cells in the ovarian stroma and endometrial stroma of uterus can be classified as TCs, given their positivity for both PDGFR-β and c-kit. Additionally, we supported our single marker analysis with double immunohistochemistry using CD34 (a membrane marker which stains the thin Tps) and α-SMA (cytoplasmic marker). These results strongly suggest that the TCLCs found in the rat ovary and uterus are indeed TCs.

Salama (2013)’s study was also involving healthy female albino rats, divided into immature, adult non-pregnant, pregnant and post-partum groups. They performed immunohistochemical identification of TCs with a c-kit antibody on the middle one-third of the right uterine horns. The number of c-kit-positive telocytes per high-power field has been calculated and statistically examined in both the endometrium and myometrium. TCs that were c-kit-positive were found in the endometrium, specifically around endometrial glands, while in the myometrium they were oriented parallel to circular smooth muscles and longitudinal muscle bundles. It should be noted that the human myometrium comprises two crucial cell populations contributing to its contractility which are smooth muscle fibres and interstitial cells. Although the pace-making mechanism remains unidentified, it is plausible that myometrial smooth muscle cells contract in response to a signal generated by c-kit-positive interstitial cells (Cretoiu et al. 2011). Given that the female reproductive system is highly hormonally regulated, TCs have been shown to exhibit different characteristics and functions during various stages of reproduction in the rat uterus (Salama 2013). For the first time, our study demonstrates the presence of TCs in the rat ovary. Therefore, we believe that investigating TCs in the rat ovary, particularly their roles across different reproductive stages, would be of significant importance for further studies.

As a conclusion, TCs, along with their Tps, Pdms and Pds, were observed throughout the stroma of the rat ovary, closely associated with follicles, corpus luteum, blood vessels, and other stromal cells. Subsequently, a meshwork of rat uterine TCs was identified in the stroma of the endometrium, myometrium and perimetrium, in close proximity to blood vessels and uterine glands. It is essential to investigate further the roles and functions of TCs in the female reproductive system as well as TC related disorders.

## Supplementary Information

Below is the link to the electronic supplementary material.

Supplementary file 1 (TIFF 6196 KB) Figure S1: Negative controls of c-kit, PDGFR-β, CD34, and α-SMA stainings for both uterus and ovary

## Data Availability

No datasets were generated or analysed during the current study.
