# Supplementary figures and images for: Distribution and morphologic characterization of telocytes in rat ovary and uterus: insights from ultrastructural and immunohistochemical analysis
Source: Histochem Cell Biol. 2024 Jul 30;162(5):373–84. doi: 10.1007/s00418-024-02313-w (PMC11393091; doi:10.1007/s00418-024-02313-w)

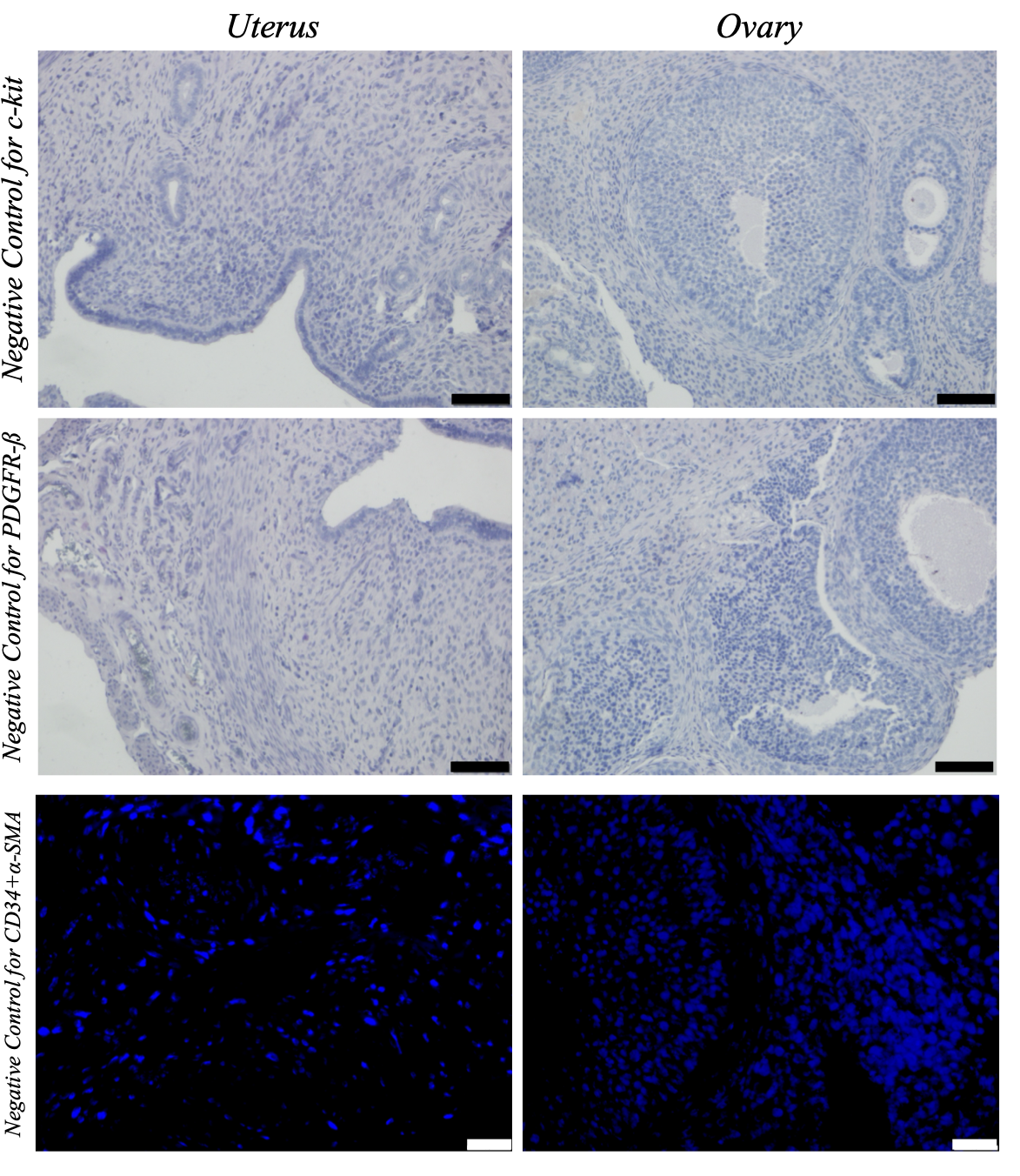

Supplement: Supplementary file 1 — (TIFF 6196 KB) Figure S1: Negative controls of c-kit, PDGFR-β, CD34, and α-SMA stainings for both uterus and ovary [file 418_2024_2313_MOESM1_ESM.tiff]
